# The Late Dr. David Hosack

**Published:** 1835

**Authors:** 


### II. ANALYTICAL.

---

### Art. I.—The late Dr. David Hosack.

The great commercial emporium of the Union, has not as yet produced a physician of more extended fame, than the able man whose name is prefixed to this article. Our earliest *medical* recollections, connect themselves more or less with his reputation. For the first quarter of the present century, but few American physicians were more incessantly occupied in the practice of the profession, in teaching and in writing. Residing at a spot so distant from the theatre of his birth and death, we cannot do better, than borrow from our cotemporary, the *Un. St. Med. and Surg. Jour.*, for Jan., 1836, the following notice, which has reached us in time for this purpose, in consequence of the delay attendant on the issuing of this number.

"The medical world, and the cultivators of the fine arts, have recently been deprived of one of their brightest ornaments, by the recent demise of the distinguished individual whose name we have placed at the head of this notice. Dr. Hosack died of apoplexy, accompanied with paralysis of the right side, after an illness of five days, on the evening of the 22d of December, ultimo, at his city residence in Chambers-street. He had just passed his 66th year.

"Dr. Hosack was born in the city of New-York, in August, 1769. His father was by birth a Scotchman; his mother, a native of New-York. Dr. Hosack received his earlier education first in Columbia College, but subsequently took the degree of A. B. at Princeton, New-Jersey. He was a private pupil in medicine and surgery under the care of the late eminent Dr. Richard Bayley. He graduated M. D. at the medical school in Pennsylvania, in 1791. Shortly after this period, he entered upon the practical duties of his profession in Alexandria, in Virginia; but the limited sphere of that place not corresponding with his wishes, he returned, after a short experiment, to his native city, and finding that many of the cultivators of the healing art, in order the better to secure a becoming patronage, had terminated their medical studies in Europe, he determined to set out for the Scottish metropolis, which then justly boasted of its far-famed university. Thither he repaired, and passed his terms at this renowned school, and formed a large circle of literary and scientific acquaintance, with whom he maintained a correspondence up to the last of his life. Upon his return to New-York, in 1795, he formed a connection in business with Dr. Samuel Bard, subsequently the second President of the College of Physicians and Surgeons in this city. Dr. Bard was eminently distinguished by his professional merits and by his widely extended practice. Upon his retirement, Dr. Hosack assumed the entire responsibility; and for a period of nearly forty years, was recognized at the head of his profession in his native place. The limits of these obituary remarks forbid us to dwell upon his peculiar characteristics as a physician; though it deserves to be stated that his practice partook largely of the anticipating method of cure: the temporizing expedients of the expectant system found little favor in his views. As a teacher of several branches of medical science, he also merits special notice. He was first appointed professor of Botany in Columbia College, in 1795, to which was shortly after added the Materia Medica, both chairs of which he held till the dissolution of the medical faculty of that institution in 1813. Upon the organization of the College of Physicians and Surgeons in the city, by the Regents of the University, tn 1807, he was appointed professor of Obstetrics and lecturer on Surgery. The former course he delivered during the first session of this new school of physic. Upon the re-organization of the College in 1813, and the union of the faculty of Columbia College with that of the College of Physicians and Surgeons, he was appointed professor of the Theory and Practice of Physic. This branch of study he continued to teach until 1826, when causes not now necessary to dwell upon, induced him, with his colleagues, to resign his appointment. Upon the establishment of the Rutgers' Medical College, he again resumed the duties of professor of the Practice of Physic and Clinical Medicine, which he fulfilled with his wonted ability until 1830—31. As a teacher of the several branches of medical science, and particularly of the practice of physic, it may be safely said that he has never had a superior in his native country, as the practical expositor of clinical medicine. The thousands of students who have witnessed the illustration of his precepts in his attendance as physician at the New-York Hospital, will bear testimony to his medical tact and efficiency.

Dr. Hosack was the author of numerous medical papers and essays; many of them monographs on particular diseases; besides occasional addresses and discourses. He was unanimously elected Fellow of the Royal Society of London, in 1816. In connection with his partner in business, and subsequently his colleague in the University, he for several years published a medical journal, the American Medical and Philosophical Register, 4 vols. 8vo. edited by David Hosack and John W. Francis. His various medical writings have been brought together in 3 vols. 8vo; in the third volume of which he has detailed at great length the vicissitudes which have marked the progress of the profession in New-York, both by individual difficulties and legislative measures. His writings on the yellow fever have been long familiarly known. He was the advocate of the specific character and contagious nature of that disease. The first edition of his Practical Nosology appeared in 8vo, in 1819. His Memoir of De Witt Clinton, in 4to, in 1828. A more disinterested tribute to the memory of a great benefactor, never was given to the public. Of his merits as associated with the New-York Historical Society, as President of the Literary and Philosophical Society of New-York, and as connected with other public institutions and private undertakings, we forbear at this time to speak. He deserves, and will doubtless receive, the just consideration of his various merits by some surviving friend, adequate to the responsible task. His remains were accompanied to their place of interment in the marble cemetry, with a regard which became so distinguished a character.

"In conclusion we may venture to say, that in the Death of Dr. Hosack the medical profession has lost one of its most substantial pillars; and that the many productions of his pen will be held in esteem side by side with those of the lamented Rush."

